# Modifying a method for human reliability assessment based on CREAM-BN: A case study in control room of a petrochemical plant

**DOI:** 10.1016/j.mex.2019.02.008

**Published:** 2019-02-12

**Authors:** Gh. A. Shirali, T. Hosseinzadeh, K. Ahamadi angali, Sh. Rostam Niakan Kalhori

**Affiliations:** a Department of Occupational Health Engineering, Faculty of Public Health, Ahvaz Jundishapur University of Medical Sciences, Ahvaz, Iran; b Department of Biostatistics and Epidemiology, Faculty of Public Health, Ahvaz Jundishapur University of Medical Sciences, Ahvaz, Iran; c Department of Health Information Management, Tehran University of Medical Sciences, Tehran, Iran

**Keywords:** Cognitive Reliability and Error Analysis Method Bastian Network, CREAM, Bayesian network, Human reliability analysis

## Abstract

**Background:**

Cognitive Reliability and Error Analysis Method (CREAM), as one of the second-generation methods, has been developed to overcome the shortcomings of the first-generation human reliability analysis methods. Although it is a useful tool for assessing the effects of context on human failure probability, namely common performance conditions (CPCs), there still exist some problems, such as lack of data about CPCs, and their unclear relationship with the operator control mode.

**Objective:**

The current paper aimed at applying CREAM Bayesian Network **(**BN) in a real-world situation in order to identify the limitations associated to CPCs in estimating Human Error Probability (HEP).

**Method:**

In this paper, the data pertaining to CPCs were collected by a self-designed questionnaire. CREAM BN was then performed in a five-step methodology, including the identification of the primary effects of CPCs, adjustment of dependency of CPCs, new grouping of CPCs, determination of control modes, and HEP calculation.

**Results:**

The results showed that there are varied values of control modes in CREAM BN in comparison with the basic CREAM. On the other hand, this method provides the grounds for incorporating various importance levels of CPCs in HEP estimation by changing the nature of prior conditional probabilities from the deterministic one into the probabilistic one.

**Conclusion:**

The methodology introduced in this study provides a simple method for the calculation of HEP in the complex industries.

• This method provides the application of the CREAM BN in a real-environmental in practice.
• This method provides a foundation for incorporating various importance levels of the CPCs in the HEP estimation by changing the nature of prior conditional probabilities from deterministic into probabilistic.
• It could reduce the uncertainty in the calculation of HEP.

## Graphical abstract

**Figure:**
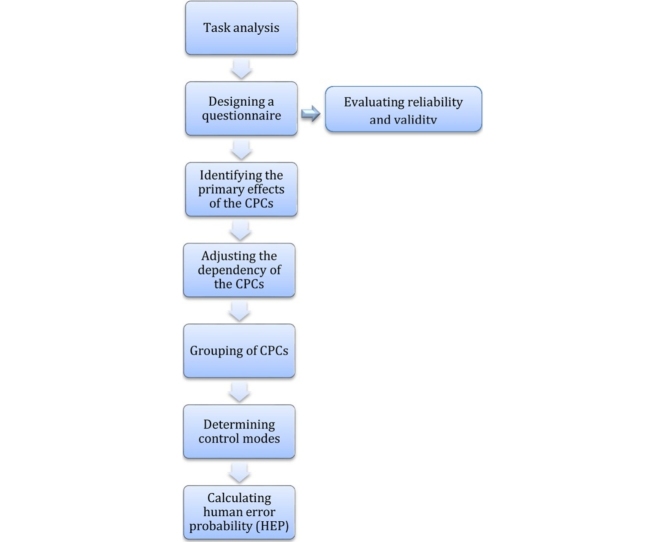

**Specifications Table**

**Table:** 

**Subject Area**	*Engineering*
**More specific subject area:**	*Cognitive Human Reliability Assessment*
**Method name:**	*CREAM BN*
**Name and reference of original method**	*Yang Z, Wang J, Rochdi M, Belkacem O, editors. Bayesian modelling for human error probability analysis in CREAM. Quality, Reliability, Risk, Maintenance, and Safety Engineering (ICQR2MSE), 2011 International Conference on; 2011: IEEE.*
**Resource availability**	*Not available*

## Method details

The tasks performed by petrochemical operators reveal a nature of highly contextual dependency where technological, environmental, and social factors often emerge and constitute a complex working condition in an interactive path [1]. In such contexts, human error can be considered as a significant factor that contributes to the risk and reliability [2,3]. Statistics show that more than 85% of the industrial accidents are attributed to the human factors [3]. Hence, human factor and, consequently, human reliability analysis [4] have always been among the significant issues and areas of interest to the risk researchers, decision-makers, safety engineers, and practitioners. Although Human Reliability Assessment [4] is subjective and human factor data are imprecise [5], it is useful for the mitigation of human errors [2]. HRA has been widely utilized in the industries, such as nuclear area, aerospace, chemical industry, petrochemical industry, and many others. The study of HRA dates back to the 1950s. After the passage of some decades from its development, a deep understanding of human error mechanism has been gained in academic circles and a number of HRA methods have been proposed in first- and second-generation modes [6].

First-generation methods, such as Technique for Human Error Rate Prediction (THERP) have been built around the pivotal assumption that, due to the inherent deficiencies, humans naturally fail to perform tasks just like a machine [2,7]. There are also some reasons why the use of first-generation methods imposes some limitations in the analysis of human factor. It can be argued that they lack a well-defined classification system, an explicit model, and an accurate representation of dynamic system interactions. Additionally, the majority of these methods specify each operator's performance as successful or failed [8]. Furthermore, performance shaping factors (PSFs), which represent the effects of the environment on human performance, are extremely weak [7,8]. To overcome the shortcomings of first-generation HRA methods and determine the relationship between the context and human failure probability, second-generation HRA methods have been developed [9]. Cognitive reliability and error analysis method (CREAM) constitutes the most common method in this group [5]. This method integrates human, technological, and organizational factors and presents a consistent error classification system. Although common performance conditions (CPCs) is a useful tool that can determine the contextual effects on human failure probability, the issues of subjectivity and lack of data about CPCs as well as the unclearness of their relation with operator control mode are still at play [2,5]. The aim of this paper is, therefore, to apply CREAM BN in a petrochemical plant so that the limitations pertaining to CPCs with regard to the estimation of HEP can be identified.

As mentioned previously, this method is one of the second-generation HRA methods that was introduced in 1998 by Hollnagel [10] and contains two versions, namely the basic and the extended ones [5]. The basic one provides an initial screening of human interaction where the tasks and its major segments are addressed by calculating the distinct sums of the positive and negative influencing CPCs use. In this regard, the relevant control mode and failure rate probability will be determined [11]. On the other hand, the extended method makes use of the results of the basic CREAM to calculate the probability of each cognitive function failure of tasks that needs more precision and detailed analysis [10]. This method has been derived from Contextual Control Mode (COCOM), which provides a conceptual and practical basis for the enhancement of operator performance [12]. Therefore, COCOM principles clarify and anticipate that human performance is the consequence of the purposive employment of a dynamic balance between human action and the system response. In this view, the essential point is that the working conditions determine human performance in system operation. The model, therefore, describes control modes in four different characteristics in accordance with human cognition and action context [1,10]. The control modes are determined by a set of nine CPCs. The four control modes include scrambled, opportunistic, tactical, and strategic states, which are linked with different failure probability intervals that represent human action failure probabilities. The nine CPCs are listed as adequacy of the organization, working conditions, adequacy of man–machine interface and operational support, availability of procedures and plans, the number of simultaneous goals, available time, time of the day, adequacy of training and experience, and crew collaboration quality [1,2,10]. In this regard, the pre-defined linguistic variables have been chosen to describe the nine CPCs. They are classified into three sets in terms of their influences (i.e., positive, negative, and neutral) on human performance reliability [1,2]. Considering the classification method, introduced by COCOM, CREAM can be used to conduct its bi-directional HRA inference, and retrospective/prospective analysis [10].

Determining the effect of the CPCs on human performance is not an easy task and is dealt with numerous uncertainties. Recently, new quantification approaches have been presented to estimate the CPCs through CREAM method [8,9]. These methods are based on fuzzy logic and provide a systematic procedure to determine the ambiguities during the quantification of CPCs so that the specific numerical values, such as human failure probabilities (HFP) can be calculated. However, they suffer from some pitfalls in practice, some of which can be listed as follows: the ignorance of the logicality of using multiple-input single-output's rule base for modeling the relations between control modes and CPCs, the loss of useful information in fuzzy Max–Min inference operations, and inadequacy of modeling CPC dependencies and of instant estimation of HFP [1]. Accordingly, BN approach has been proposed [9] to deal with CPC dependencies [1]. In this context, BN, as one of the most efficient models, is utilized in the fields with uncertain knowledge and reasoning [13]. BN is a graphical model for reasoning under uncertainty where the nodes represent variables (discrete or continuous) and the arcs represent the direct connections between them. It also represents the conditional dependencies of a set of random variables through a directed acyclic graph [14,15]. Hence, the relationship between the CPCs and control modes can be modeled both realistically and systemically in COCOM by using BN.

There is a wealth of research on the quantification of CPCs’ values [1,4,9,15] wherein the systematic procedures have been proposed to account for the ambiguity in the quantification of CPCs’ values as outputs for HEP estimation. Most of the proposed approaches were developed and tested in a laboratory context. In addition, it is probable that analysts encounter the incidence of some error in such a process as the assessment of the state of each CPC is often very difficult and time-consuming. Therefore, the authors integrated the two methods proposed by Yang et al. [1,15] and practically implemented the integrated method in a real environment. For example, in the current study, a self-disigned tool was used to collect data pertinent to the CPCs. This tool, indeed, may facilitate data collection relating to the CPCs. The authors also tried to simplify the mentiond method as far as possible and, thereby, the collection and analysis of information and data pertaining to the CPCs became much easier than those of Yang’s methods. Therefore, the differences between the current study and the previous studies lie in the data collection process, the context or environment of the study, and the method simplicity.

### Context of study

Petrochemical industries are characterized by very high levels of risks due to the nature of work processes and dangerous substances used in these installations. Hence, the incidence of human error in these industries will not only affect the personnel and facilities but it can also result in a catastrophic consequence beyond the boundaries of the plant [16,17]. Statistics demonstrate that human error is a major contributor in over 80% of chemical and petrochemical accidents [18]. In this regard, the olefin unit (due to its risks) of one of the petrochemical industries in south-west of Iran was selected as the target of this study. The number of 62 employees was working in four shifts (the work shift plus the rest shift) in this unit. In fact, each of the employees worked about 8 h a day. The number of the employees per shift and their distribution are presented as follows: head of the unit (1 person), shift control (2 people), shift supervisor (1 person), board-man operators (6 people), and outside operators (8 people). On the other hand, there were three operational shifts with 54 people and a rest shift with 8 people.

## Methodology

This study attempts to introduce the application of a method on HRA (based on CREAM BN being derived from the previous studies) in a petrochemical plant. In this study, the basic version of CREAM was modified according to Yang’s methods and implemented in a real environment. The research steps and the stages of change made on Yang’s methods are presented as follows:

### Data collection

The data relating to the CPCs were gathered via a self-designed questionnaire whose questions were developed to cover 9 CPCs (measuring dimensions) (Table 1). Therefore, the questionnaire was designed with 70 questions, which were scored based on five-point Likert scale from “strongly disagree” to “strongly agree”. Likert scale was used to evaluate the employee's opinions about a specific statement which represents the level of agreement or disagreement about the CPCs. There are different kinds of likert scales including; 5 point likert scale, 7 point likert scale or 9 point likert scale [19,20]. An oral negotiation with the operators revealed that responding to questions through five-point scales would be much easier than responding to items through seven-point Likert scales. The reliability and validity of the instrument were also evaluated. For example, some of the questions about the nine CPCs, which were raised to the employees, are separately presented as follows:

• Adequacy of organization. This CPC was evaluated through ten questions, such as “The employees were selected to perform their job according to the individual competence, experience, physical, and mental ability.”
• Working conditions. This CPC was evaluated through eight questions, such as “Noise pollution causes disturbance in the speech communication and work performance of the employees.”
• Adequacy of MMI (man–machine interface) and operational support. This CPC was evaluated through eight questions, such as “Display information are readily and easily available and understandable to the employees in the workplace.”
• Availability of procedures/plans. This CPC was evaluated through sixteen questions, such as “Access to procedures/plans is as easy as possible for the employees in the workplace.”
• Crew collaboration. This CPC was evaluated through six questions, such as “Interaction and collaboration are highly desirable among the employees in the workplace.”
• Available time. This CPC was evaluated through five questions, such as “The available time for task performance leads to the employees' fatigue in the workplace.”
• Time of the day. This CPC was evaluated through four questions, such as “Shift work causes fatigue of the employees and reduces productivity performance in the workplace.”
• Adequacy of training and experience. This CPC was evaluated through ten questions, such as “Need assessment of the training programs is accomplished according to the characteristics of trainees in the workplace.”
• Number of simultaneous goals. This CPC was evaluated through four questions, such as “The employees have to pay attention to many factors to accomplish the tasks in the workplace.”

**Table 1:** CPCs and performance reliability [14].

CPC		CPC level/description	Expected effects on performance
#1	Adequacy of organization	*Deficient (L_1,1_)*	Negative
		*Efficient (L_1,2_)*	Neutral
		*Inefficient (L_1,3_)*	Negative
		*Very efficient(L_1,4_)*	Positive
#2	Working conditions	*Incompatible (L_2,1_)*	Negative
		*Compatible (L_2,2_)*	Neutral
		*Advantageous (L_2,3_)*	Positive
#3	Adequacy of MMI and operational support	*Inappropriate (L_3,1_)*	Negative
		*Tolerable (L_3,2_)*	Neutral
		*Adequate (L_3,3_)*	Neutral
		*Supportive (L_3,4_)*	Positive
#4	Availability of procedures/plans	*Inappropriate (L_4,1_)*	Negative
		*Appropriate (L_4,2_)*	Neutral
		*Acceptable (L_4,3_)*	Positive
#5	Number of simultaneous goals	*More than capacity (L_5,1_)*	Negative
		*Matching current capacity (L_5,2_)*	Neutral
		*Fewer than capacity(L_5,3_)*	Neutral
#6	Available time	*Continuously inadequate (L_6,1_)*	Negative
		*Temporarily inadequate (L_6,2_)*	Neutral
		*Adequate (L_6,3_)*	Positive
#7	Time of the day	*Night-time (unadjusted) (L_7,1_)*	Negative
		*Day-time (adjusted) (L_7,2_)*	Neutral
#8	Adequacy of training and experience	*Inadequate (L_8,1_)*	Negative
		*Adequate, limited experience (L_8,2_)*	Neutral
		*Adequate, high experience (L_8,3_)*	Positive
#9	Crew collaboration	*Deficient (L_9,1_)*	Negative
		*Efficient (L_9,2_)*	Neutral
		*Inefficient (L_9,3_)*	Neutral
		*Very efficient (L_9,4_)*	Positive

The reason for selecting nine CPCs is that the analyst describes the effect of work conditions (context) on human error in the jobs under analysis. Due to working conditions in the mentioned industry and errors occurred, the research team selected 9 CPCs to examine the context effects on human error. Moreover, the base of the CREAM was established on these nine CPCs.

Data collection via this method is much easier than the ones proposed in the previous studies. For example, one of its merits is the collection of complete and accurate information about the CPCs in a short time by a few experts rather than an expert. Therefore, researchers can analyze many jobs or tasks in a short period by this method. Moreover, since the level of each CPC (presented in Table 1) is evaluated by several and different questions in this method; uncertainty and bias will be decreased in the final score of each CPC. Accordingly, we can say that the collected information about each CPC has an acceptable accuracy.

To test the reliability of the CREAM BN method, the specified control modes in Bayesian and basic CREAM were compared in four jobs, i.e., head of control, shift supervisor, Boardman operator, and outside operator.

### HEP calculation

For HEP calculation, the following steps should be conducted:

• Identifying the primary effects of the CPCs: As presented in Table 1, each CPC consists of the limited linguistic terms (2, 3 or 4) regarding the expected effects on performance reliability wherein each term is associated with a primary effect on HEP. The positive effects lead to an increase in human reliability but the negative ones do the opposite. The main effects of each CPC are considered as a useful tool for the estimation of HEP in Bayesian network. However, the effect of each CPC on the COCOM_s_ in the basic CREAM is both discrete and deterministic (either 0% or 100%), and the failure rate intervals are too wide; therefore, this study used fuzzy numbers to convert discrete and deterministic values to continuous values (Table 2). In this way, it was made possible to incorporate various importance levels of the CPCs in HEP estimation and reduce the uncertainty. This step was also pointed to in the existing CREAM BN methods, but we tried to simplify it. In other words, identifying the primary effects of the CPCs is very simple in the current study. For this purpose, the CPCs’ information about the discrete and deterministic domains was gathered using the self-designed tool and was then converted to continuous values according to Table 2. This process, in contrast to what was presented in the previous studies could reduce the subjectivity to some extent. On the other hand, the studies based on CREAM BN are able to incorporate uncertainty in estimating CPCs and therefore to reduce subjectivity in this step. While the previous studies cannot established a strong correlation between the levels of nine CPCs and four control modes. Therefore, it can be a kind of uncertainty.

**Table 2:** Fuzzy set supports [7].

	CPC	Membership level interval			
1	Adequacy of organization	Deficient	Inefficient	Efficient	Very efficient
		(0–25)	(10–60)	(40–90)	(70–100)
2	Working conditions	Incompatible	Compatible	Advantageous	
		(0–30)	(20–80)	(70–100)	
3	Adequacy of MMI and operational support	Inappropriate	Tolerable	Adequate	Supportive
		(0–25)	(10–60)	(40–90)	(70–100)
4	Availability of procedures	Inappropriate	Acceptable	Appropriate	
		(0–30)	(20–80)	(70–100)	
5	Number of simultaneous goals	More than capacity	Matching current capacity	Fewer than capacity	
		(0–30)	(20–80)	(70–100)	
6	Available time	Continuously inadequate	Temporarily inadequate	Adequate	
		(0–30)	(20–80)	(70–100)	
7	Time of the day	Night	Day	Night	
		(0–11)	(8–20)	(16–24)	
8	Adequacy of training and experience	Inadequate	Adequate, limited experience	Adequate, high experience	
		(0–30)	(20–80)	(70–100)	
9	Crew collaboration	Deficient	Inefficient	Efficient	Very efficient
		(0–25)	(10–60)	(40–90)	(70–100)
• Adjusting the dependency of the CPCs: Unlike the first-generation HRA methods, the CPCs (in CREAM) due to their effects on human performance reliability may depend on each other. This means that if a CPC has a neutral effect on the context and be dependent upon other CPCs, then the effect of this CPC may undergo some changes towards either a positive or negative direction based on conditions of the other CPCs. Therefore, the rules governing these changes, as defined by Hollanagel [10], were used to determine the degree of dependency among the CPCs. Table 3 reflects such changes. According to Table 3, CPC_2, 5, 6, 9_ are adjusted (principal) CPC and CPC_1, 3, 4, 7, 8_ are unadjusted (dependent) CPC. For example, if CPC_9_ (a principal CPC) is efficient (neutral), CPC_1_ (its dependent CPCs) is deficient (negative), CPC_8_ is inadequate (negative), and it does not matter which linguistic variables are utilized to describe the other CPCs, then CPC_9_ will be adjusted as deficient (negative) rather than efficient (neutral) ([10]).

**Table 3:** Rules for adjusting CPCs [9].

Principal CPC	Dependent CPCs				
CPC_2_	CPC_1_	CPC_3_	CPC_6_	CPC_7_	CPC_8_
CPC_5_	CPC_2_	CPC_3_	CPC_4_	CPC_5_	CPC_7_
CPC_6_	CPC_2_	CPC_3_	CPC_4_		
CPC_9_	CPC_1_	CPC_8_
• New grouping of CPCs: Since three of the CPCs contain 4 levels, five of them include 3 levels, and one of them has 2 levels, 31,104 (4^3^*3^5^*2) conditional probabilities were assessed to build a network. This was an extremely difficult task, but it was feasible in practice. Therefore, there are two ways for reducing conditional probabilities required. One is to use positive and negative effect nodes to model CPCs levels and reduce conditional probabilities in two separate sets. The other is to reduce the number of parent nodes using a divorce approach [15]. The CPCs were classified in three groups based on the second approach:
• G1: CPC_1_, CPC_2_, CPC_3_
• G2: CPC_4_, CPC_5_, CPC_6_
• G3: CPC_7_, CPC_8_, CPC_9_

This classification does not have any physical meaning and is used only to reduce the software load.

Conditional probabilities were determined according to Yang’s et al. [15] study. The kernel of this study is to appropriately transform belief degrees in rule bases into the conditional probabilities in Bayesian network [15]. The rule base with the belief structures is firstly depicted in the form of the conditional probabilities. For this purpose, the discrete functions *f*_Ei,_ *f*_A_, *f*_Gl_, *f*_G_ and *f*_COCOM_ should be defined in the first step. *f*_Ei_ determines the primary effect of the CPC from the level of the CPC. *f*_Ei_ can be defined as below:

(1) $$f_{Ei\;}:\;{CPC}_{i\;}\to E_{i}(\text{i}=1,2,\;\ldots ,\;9)\;$$

E is the set of expected effect (negative, natural, positive) on performance reliability as shown in Table1. Based on Eq. (1), the conditional probability table of primary effect can be determined according to Table 4.

**Table 4:** Conditional probability table corresponding to the function f_Ei_.

Level of CPC_1_	Expected effect (E)		
	Positive	Neutral	Negative
Very efficient	1	0	0
Efficient	0	1	0
Inefficient	0	0	1
Deficient	0	0	1

Table4. Conditional probability table corresponding to the function f_Ei_

In the second step, the rules presented in Table 5 are used for determining the node adjusted effect or deterministic conditional probability function *f*_A_ as follows:

(2) $$f_{Ah}:\left(E_{h}+E_{h,D}\right)\to A_{h}\;(h=\#2,\;\#5,\;\#\;6,\;\#9)$$

*E*_h_ shows the natural effect of a principal CPC; *E*_h,D_ indicates the positive or negative effect of its dependent CPCs; *A*_h_ means the adjusted positive or negative effect of the principal CPC. For instance, the conditional probability of CPC_9_ (*f*_A#9_) is presented in Table 5.

**Table 5:** Conditional probability table corresponding to CPC#9 (*f*_A#9_).

*E_h_*	*E_h,D_*		*A_h_*	
CPC#9	*CPC#1*	*CPC#8*	*CPC#9*	
Y^a^	Y	Y	Y	N
		N	1	0
	N	Y	0	1
		N	0	1
N^b^	Y	Y	0	1
		N	0	1
	N	Y	0	1
		N	0	1

a Yes.

b No.

In the next step, we should define the discrete functions *f*_Gl_ and *f*_G_ by counting the number of the positive/negative effects, respectively. Then, the CPC levels presented in Table 1 are used to develop G_l_ and G as follows:

G_l_ = (3 effects, 2 effects, 1effects, 0effects)

G = (9 effects, 8 effects, 7 effects, 6 effects, 5 effects, 4 effects, 3 effects, 2 effects, 1 effects, 0 effects)

G_l_ and G should be modified because Table1 indicate that CPC#5 and CPC#7 have no positive effect; therefore it can be written:

G_positive,l_ = (2 effects, 1effects, 0effects) (l = 2 and 3)

G_positive_ = (7 effects, 6 effects, 5 effects, 4 effects, 3 effects, 2 effects, 1 effects, 0 effects)

However, the conditional probabilities related to *f*_Gl_ and *f*_G_ using positive effects are presented in Table 6, Table 7.

**Table 6:** Conditional probability table related to *f*_Gl_.

Positive effect			Positive effect G_1_			
1	2	3	3 effects	2 effects	1 effects	0 effects
Y	Y	Y	1	0	0	0
		N	0	1	0	0
	N	Y	0	1	0	0
		N	0	0	1	0
N	Y	Y	0	1	0	0
		N	0	0	1	0
	N	Y	0	0	1	0
		N	0	0	0	1

**Table 7:** Conditional probability table related to *f*_G_.

Positive effect			Positive effects							
G_1_	G_2_	G_3_	7	6	5	4	3	2	1	0
3 effects	2 effects	2 effects	1	0	0	0	0	0	0	0
3 effects	2 effects	1 effects	0	1	0	0	0	0	0	0
3 effects	2 effects	0 effects	0	0	1	0	0	0	0	0
3 effects	1 effects	2 effects	0	1	0	0	0	0	0	0
…	…	…	…	…	…	…	…	…	…	…

**Fig. 1:**
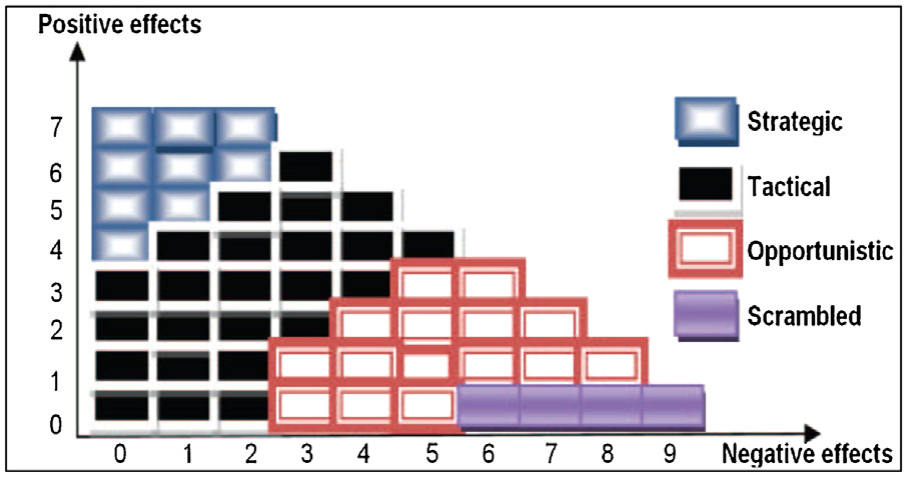
Relations, CPCs scores, and control modes [1].

Finally, we should define *f*_COCOM_ using Fig. 1.The conditional probabilities corresponding to COCOM_j_ are presented in Table 8.

**Table 8:** Conditional probabilities related to *f*_COCOM_.

Positive effects	Negative effects	COCOM_s_			
		Strategic	tactical	Opportunistic	Scramble
0	0	0	1	0	0
0	1	0	1	1	0
0	2	0	1	1	0
0	3	0	0	1	0
…	…	…	…	…	…

The evidential reasoning was used for calculating the conditional probabilities as was introduced by Yang et al. [1]. The core of this study is to establish the fuzzy IF-THEN rule bases with the belief structure. The aim of these rule bases is to recognize suitable significances for each linguistic term in the antecedent and identification of appropriate terms in the outcome. In this context, the effects stem from the membership functions of linguistic terms established by expert can be reasonably took into account [1,2]. Fuzzy rule bases (in CREAM) were modeled using a Bayesian Network technique into ten node converging connection as shown in Eq. (3).

(3) $$\begin{array}{cc} P\left(COCOM_{j\;}\right)=\prod_{i=1}^{N}P\left(COCOM_{j\;}\text{|}N_{i\;J_{i\;}}\right)P(N_{i\;J_{i\;}})\; & \left(i=1,\;\ldots ,\;9;j=1,\;\ldots ,\;4\right)\; \end{array}$$

**Fig. 2:**
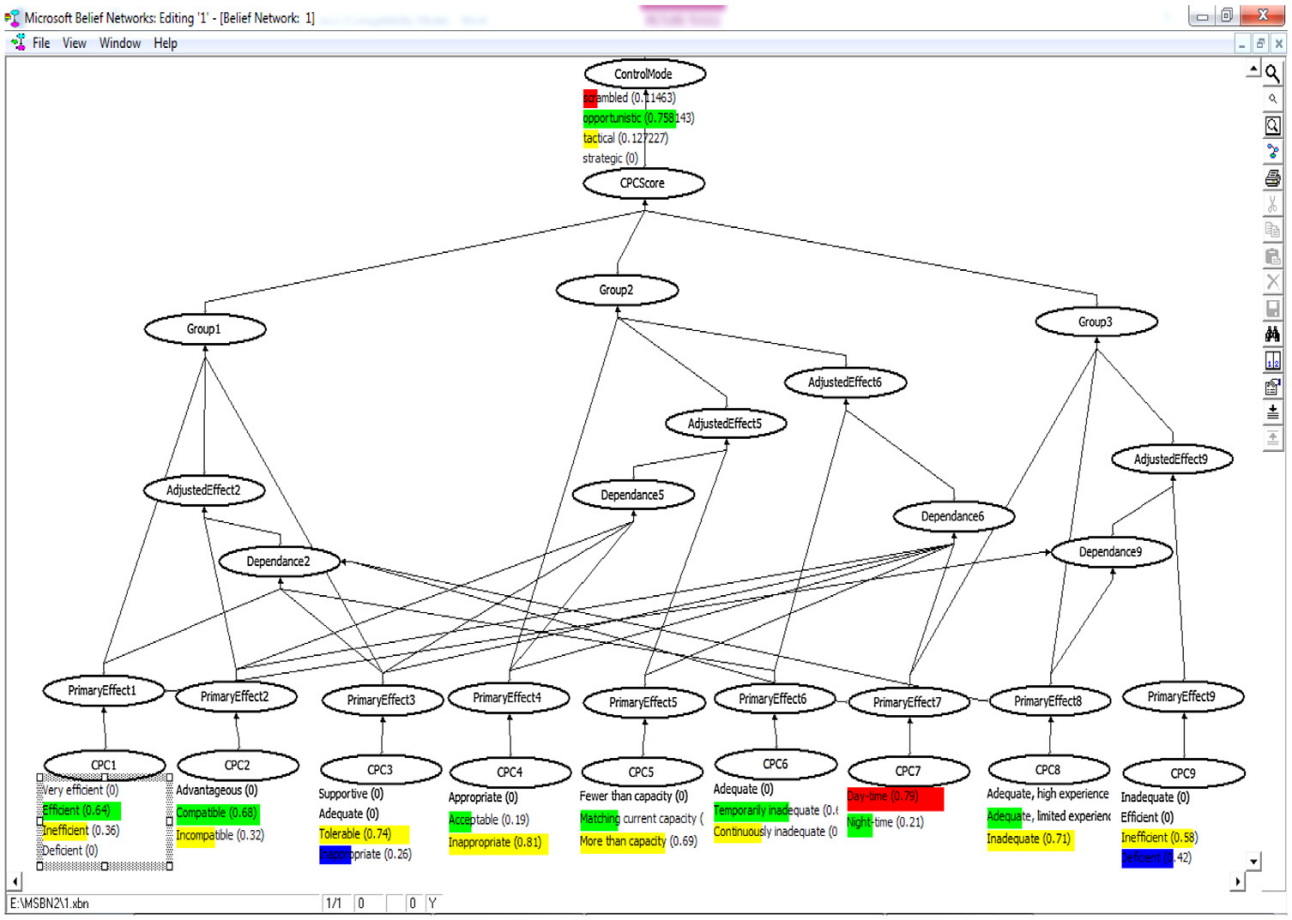
Bayesian network defined for the head control.

**Fig. 3:**
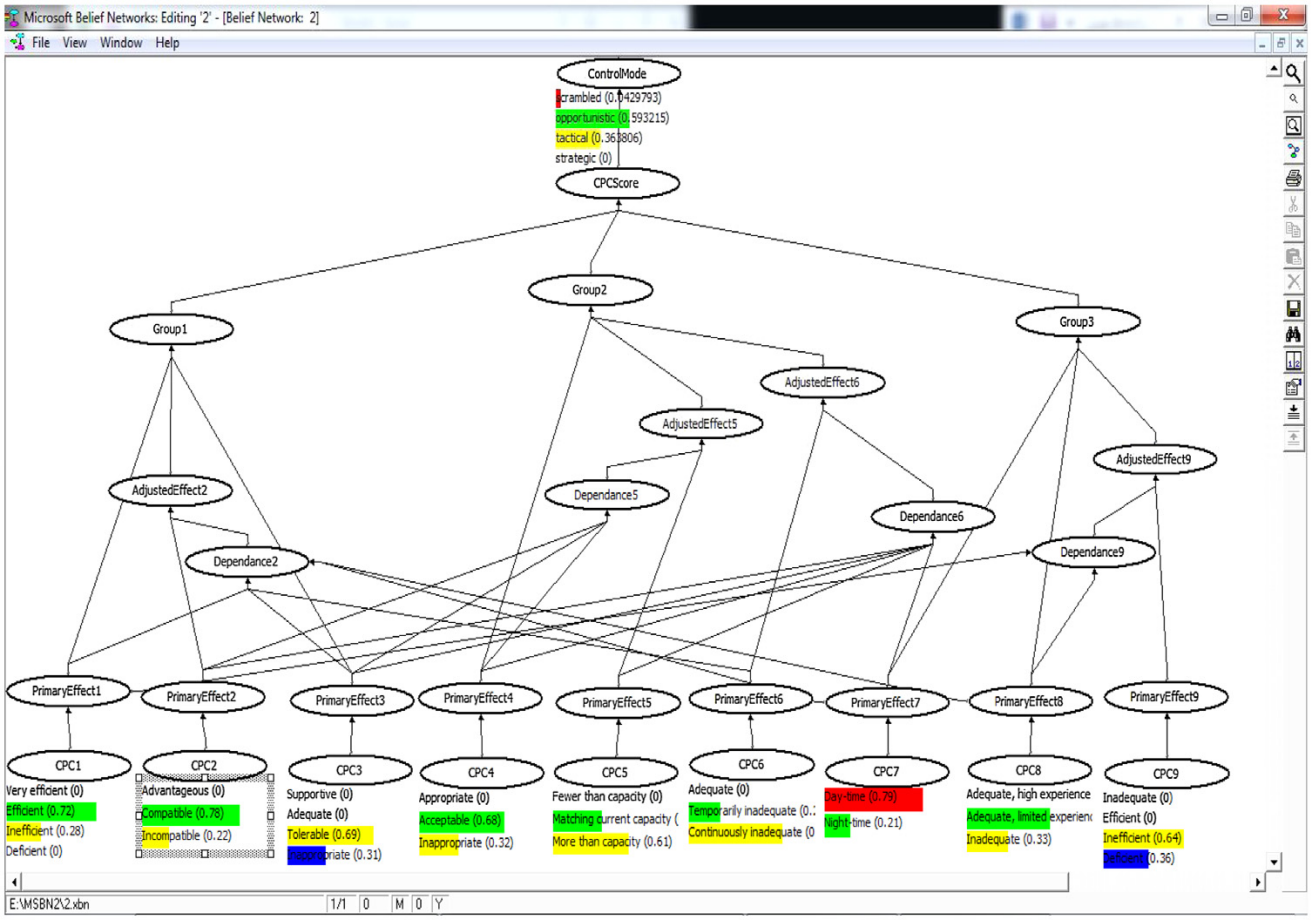
Bayesian network defined for the supervisor control.

**Fig. 4:**
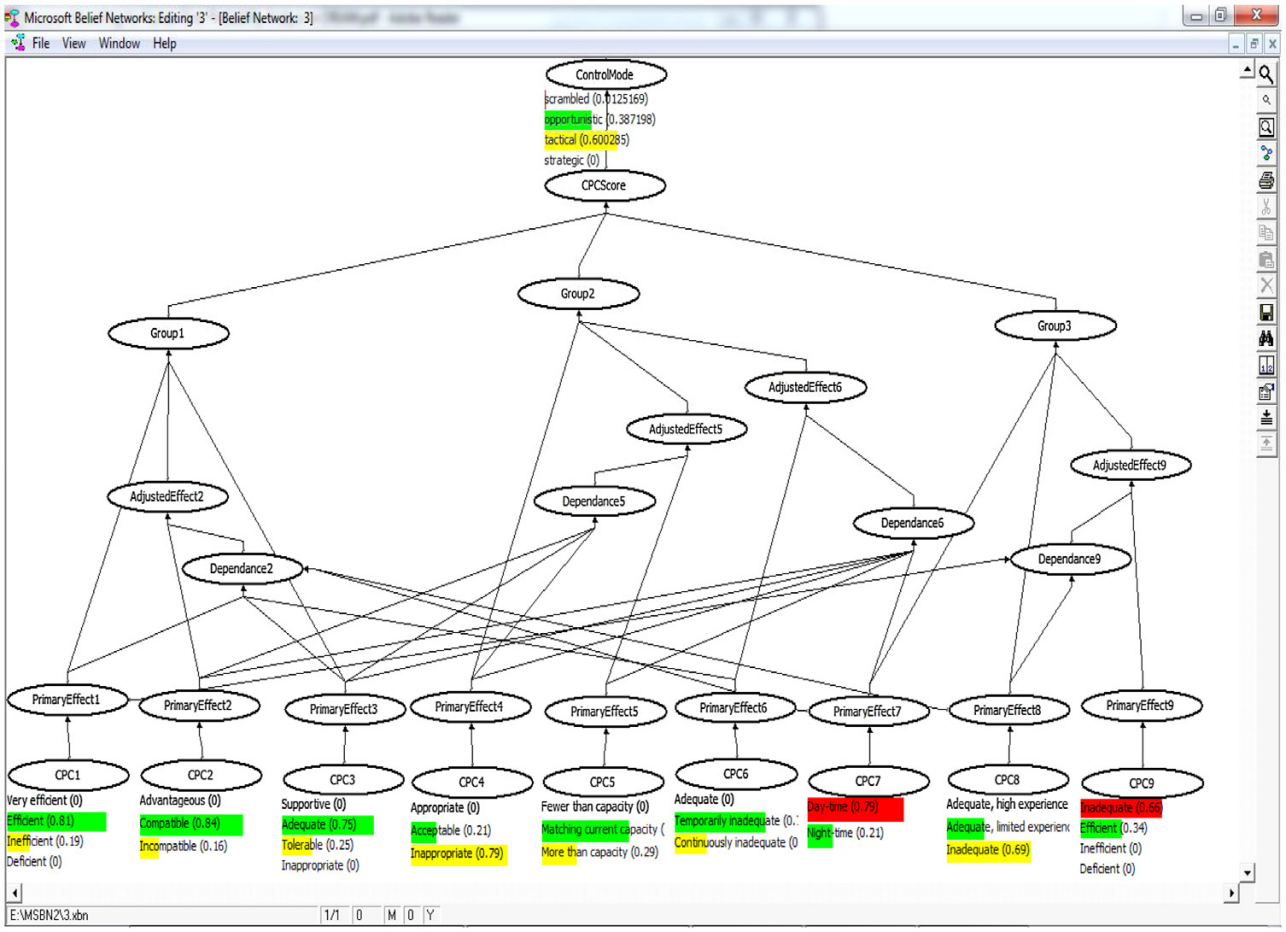
Bayesian network defined for the Boardman operator.

**Fig. 5:**
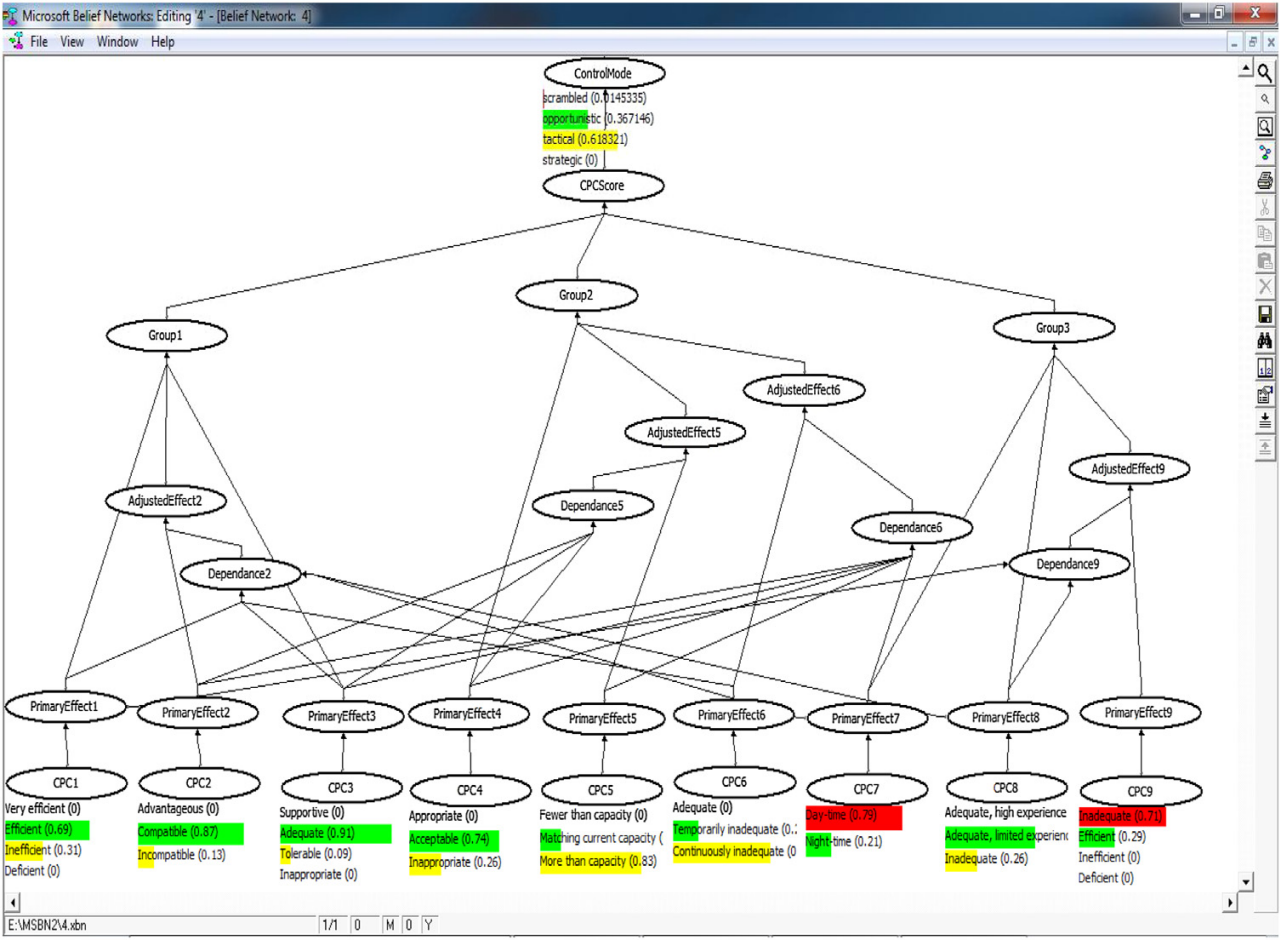
Bayesian network defined for the outside operator.

COCOMj: The probability of human action reliability related to the j^th^ control mode in Fig. 2, Fig. 3, Fig. 4, Fig. 5. P (COCOMj│N_iJi_) can be calculated using the established rule base and Eq. (4) as follows:

(4) IF L_1,3_ AND L_2,1_ AND L_3,1_ AND L_4,1_ AND L_5,1_ AND L_6,1_ AND L_7,1_ AND L_8,1_ AND L_9,1_, THEN the probability of COCOMj (j = 1, 2, 3, 4) is (0,0,0.5,0.5) or p(COCOMj│L_1,3_,L_2,1_,L_3,1_,L_4,1_,L_5,1_,L_6,1_,L_7,1_,L_8,1_,L_9,1_)= (0,0,0.5,0.5)

L _(i_,_j_): level of CPCs obtained from Table 1 (i = 1, 2, …, 9) and (j = 1, 2, 3, 4)

N_iJi_: Nine parent nodes (N_i_, i = 1, 2,…, 9) representing the nine newly adjusted CPCs and one child node associated with control modes (Ji = 1, 2, …,4). P (N_iJi_) can be calculated using the Bayesian network based on adjustment rules in Fig. 2, Fig. 3, Fig. 4, Fig. 5. See more in [1].

Where “│’’ symbolizes the conditional probability.

• Determining control modes: The core concept of calculating HEP in CREAM is the control mode [15]. The probable control modes in the basic CREAM method are determined using the CPCs' values. More specifically, the relationship between the CPCs values and the control modes is obtained from Fig. 1 where the x and y axes represent the sum of the linguistic labels of the antecedents with negative and positive effects on human reliability. In this context, 52 conditional probabilities were directly obtained from Fig. 1 and 28 ones were found to be in need of further analysis based on the inherent logic in the control mode diagram. For example, p (COCOM│Posetive effect = 7, Negative effect = 2) is not available in the diagram [15]. In this context, the analyst should ignore the evaluation of the relationships between CPCs and COCOMs using rule bases or estimate the subjectively of them. In the current study (or the studies based on Bayesian Network), this relationship between CPCs and COCOMs were objectively calculated with different effects as input weights. All calculations in this step were performed by non-commercial MSBNx software, which can be downloaded at http://research.microsoft.com/adapt/MSBNx/.
• Calculating the human error probability (HEP): Finally, total HEP for the specific actions, head of unit, shift control, shift supervisor, board-man, and outside operator were calculated using the results obtained from the above-mentioned steps. In this context, HEP value for each job can be calculated according to Eq. (5) [15].

(5) $$HEP=\;\sum_{j=1}^{4}p({COCOM}_{j})U_{{COCOM}_{j}}\;$$

Where p (${COCOM}_{j})$ represents the probability of the human action reliability belonging to the j^th^ control mode in COCOM node and $U_{{COCOM}_{j}}$ indicates utility values. Review of the related literature [6,8] indicates that the set of values, {2.24 × 10^−4^, 0.01, 0.0708 and 0.316} is employed to describe $U_{{COCOM}_{j}}$ (j = 1, …, 4} for a benchmarking purpose.

J = 4,3,2,1 (scramble, opportunistic, tactical, strategic)

## Results

### Validity and reliability of the questionnaire

As it was mentioned earlier, the data pertaining to this section were gathered by a self-designed questionnaire including 70 questions. The questions were constructed to cover 9 CPCs, as presented in Table 1.

Content validity of the questionnaire was evaluated based on the Lawshe method. In this line, the questionnaire was submitted to 10 experts to comment on the content. The results were revealed that the content validity ratio (CVR) for all questions exceeded 0.62 (the minimum acceptable value based on the number of experts). Moreover, CVR was obtained equal to 0.72 for the entire questionnaire.

The internal consistency of the responses was measured by the Cronbach's alpha coefficient where the value of 0.78 was obtained for the whole questionnaire. This value is generally regarded as an acceptable reliability level.

### Results of CPCs assessment

The results of each action derived from the questionnaire, filled out by 60 employees (two employees were not returned the questionnaire), are presented in Table 9. The values given below for each CPC in Table 9 are the primary scores that are extracted from the questionnaire. These scores are converted to the fuzzy values using the fuzzy numbers (Table 2) in order to overcome the shortcomings existing in the traditional deterministic CREAM method. The CPCs were rated from zero to hundred [0,100] except for the CPC “Time of day” that is [0, 24]. In this context, defuzzification was performed based on two ways: mean of maximum and center of area method, see more in [7].

**Table 9:** CPC_s_ scores derived from questionnaire in the various jobs.

Job	CPC_1_	CPC2	CPC3	CPC4	CPC5	CPC6	CPC7	CPC8	CPC9
Head of control	53.4 ± 6.5	41.2 ± 7.4	62.2 ± 7.5	36.1 ± 6.4	11.7 ± 5.8	16.5 ± 4.4	14.0 ± 0.5	37.4 ± 5.1	87.2 ± 7.8
Shift supervisor	60.2 ± 7.4	39.9 ± 6.2	59.4 ± 7.1	15.3 ± 5.6	28.5 ± 4.2	36.2 ± 5.1	14.0 ± 0.5	28.5 ± 4.4	85.7 ± 5.7
Boardman operator	55.6 ± 6.6	35.4 ± 5.7	20.3 ± 6.8	33.5 ± 4.7	20.2 ± 5.4	18.1 ± 6.4	14.0 ± 0.5	35.1 ± 4.7	22.4 ± 6.9
Outside operator	52.7 ± 5.2	31.1 ± 7.3	23.1 ± 5.4	13.6 ± 3.5	17.3 ± 4.9	32.4 ± 5.6	14.0 ± 0.5	19.5 ± 3.9	20.8 ± 5.1

### Analysis of Bayesian network for HEP calculation

The fuzzy values (Table 2) were processed by MSBNx software to determine the control modes and HEP. Fig. 2, Fig. 3, Fig. 4, Fig. 5 show the results of Bayesian analysis for determining the control mode. The conditional probability table is used for the node effects, the node adjusted effects, CPC scores and control modes in Fig. 2, Fig. 3, Fig. 4, Fig. 5. In simple terms, the Figs. show the relation and dependency among CPCs and their effects on the COCOM. The results pertaining to CPCs, control modes, and HEP values are presented in Table 10. The data presented in this table are composed of two parts. The first part refers to the deterministic CPCs level, control modes, and the HEP associated values, which were determined based on the basic CREAM. The second part is, however, related to the semi-deterministic CPCs levels based on the fuzzy logic, control modes, and the HEP values, which were determined based on Bayesian CREAM. A blunt difference is obvious between information in part 1 and 2. On the other hand, the calculations based on Bayesian CREAM are much more varied than what can be derived from the basic CREAM. The values belonging to CPCs levels are qualitative and deterministic in the basic CREAM, while these values are quantitative and semi-deterministic in Bayesian CREAM.

**Table 10:** The results of CPCs level, control modes, and HEP values for basic CREAM and Bayesian CREAM.

	CPC	CPC#1	CPC#2	CPC#3	CPC#4	CPC#5	CPC#6	CPC#7	CPC#8	CPC#9
Head of control	Deterministic CPC level	Efficient	Compatible	Adequate	Acceptable	More than capacity	Continuously inadequate	Daytime	Adequate, limited experience	Very efficient
	Basic CREAM	0Scramble	0 Opportunistic	1Tactical	0Strategic	HEP = 0.01				
	Semi-deterministic CPC level based on fuzzy logic	(0.3Ine) (0.7 Eff)	(0.1Inc) (0.9Com)	(0.1Tol) (0.9Ade)	(0.3Ina) (0.7Acc)	(0.8Mor) (0.2Mat)	(0.8Con) (0.2Tem)	(0.2Nig) (0.8Day)	(0.2Nig), (0.8Day)	(0.3Eff) (0.7Very)
	Bayesian network	0.0145Scramble	0.3671Opportunistic	0.6183Tactical	0Strategic	HEP = 0.03				
Shift supervisor	Deterministic CPC level	Efficient	Compatible	Adequate	Inappropriate	Matching current capacity	Temporarily inadequate	Daytime	Inadequate	Very efficient
	Basic CREAM	0Scramble	0 Opportunistic	1Tactical	0Strategic	HEP = 0.01				
	Semi-deterministic CPC level based on fuzzy logic	(0.2Ine) (0.8 Eff)	(0.2Inc) (0.8Com)	(0.3Tol) (0.8Ade)	(0.8Ina) (0.2Acc)	(0.3Mor), (0.7Mat)	(0.3Con), (0.7Tem)	(0.2Nig) (0.8Day)	(0.7Inad), (0.3Ade)	(0.3Eff) (0.7Very)
	Bayesian network	0.0125Scramble	0.3872Opportunistic	0.6003Tactical	0Strategic	HEP = 0.03				
Boardman	Deterministic CPC level	Efficient	Compatible	Adequate	Acceptable	More than capacity	Continuously inadequate	Daytime	Adequate, limited experience	Inefficient
	Basic CREAM	0Scramble	2Opportunistic	Tactical	0Strategic	HEP = 0.02				
	Semi-deterministic CPC level based on fuzzy logic	(0.3Ine) (0.7Eff)	(0.2Inc) (0.8Com)	(0.7Tol) (0.3Ina)	(0.3Ina), (0.7Acc)	(0.6Mor), (0.4Mat)	(0.7Con), (0.3Tem)	(0.2Nig) (0.8Day)	(0.3Inad), (0.7Ade Lim)	(0.4Def) (0.6Ine)
	Bayesian network	0.0430Scramble	0.5932Opportunistic	0.3638Tactical	0Strategic	HEP = 0.08				
Out-side operator	Deterministic CPC level	Efficient	Compatible	Adequate	Inappropriate	More than capacity	Temporarily inadequate	Daytime	Inadequate	Inefficient
	Basic CREAM	0Scramble	2Opportunistic	Tactical	0Strategic	HEP = 0.01				
	Semi-deterministic CPC level based on fuzzy logic	(0.3Ine) (0.7 Eff)	(0.3Inc) (0.7Com)	(0.7Tol) (0.3Ina)	(0.8Ina) (0.2Acc)	(0.7Mor), (0.3Mat)	(0.4Con), (0.6Tem)	(0.2Nig) (0.8Day)	(0.7Inad), (0.3Ade Lim)	(0.4Def) (0.6Ine)
	Bayesian network	0.1146Scramble	0.7581Opportunistic	0.1272Tactical	0Strategic	HEP = 0.09				

**Ine**: Inefficient; **Tol:** Tolerable; **Mor:** More than capacity; **Nig:** Night-time; **Ade Lim:** Adequate, limited experience inadequate; **Eff:** Efficient; **Ade:** Adequate; **Mat:** Matching current capacity; **Day:** Day-time; **Def:** Deficient; **Inc:** Incompatible; **Ina:** Inappropriate; **Con:** Continuously inadequate; **Inad:** Inadequate; **Com:** Compatible; **Acc:** Acceptable ; **Tem:** Temporarily inadequate ; **Very:** Very efficient.

Finally, Table 10 shows the values of CPCs, control modes and HEP in the basic and Bayesian CREAM among different jobs. These values can be used to evaluate the reliability of the method. As shown in Table 10, the dominant control mode among the different jobs is the same in two basic and Bayesian CREAM. For example, the dominant control mode in the both basic and Bayesian CREAM is “Tactical”. In addition, the produced result of HEP in this job is almost equal with the basic and Bayesian CREAM.

## Discussion

Human error in HRA and its quantification with respect to the qualification of the contextual scenario in which the human action is performed are among the crucial problematic issues in this domain. Due to the intrinsic difficulty of performing this evaluation and the lack of collected data, the choice of a CPC level (basic method) is affected by uncertainty. In this study, the contextual evaluation was fulfilled using CREAM BN to capture this uncertainty in a real-world setting. However, a more detailed discussion about the obtained results is presented here. The results of Table 10 show that two CPCs out of the nine CPCs, i.e., CPC_5_ and CPC_6_ have a negative effect on performance reliability of the head control operator. On the other hand, as previously mentioned in Table 3, since CPC_2, 3, 4_ are dependent on CPC_5_, and CPC_2, 3, 4, 5, 7_ are dependent upon CPC_6_; the negative effect of independent CPC_5,6_ will be influenced. Reviewing job descriptions and having conversations with the operators, the researchers identified that the operators in some cases (e.g. emergencies) have to do several simultaneous tasks (CPC_5_) in a short time (CPC_6_). Performing several simultaneous tasks in an inadequate amount of time can affect other CPCs, such as “working conditions”, “adequacy of MMI and operational support”, and “availability procedures”, as well. In such a context, the probability of the occurrence of the operator error can experience an increase and the system may be faced with a serious challenge. However, the probability distribution of the control mode is varied for the head control operator so that scramble (0.01), opportunistic (0.37), and tactical variations (0.62) were put in this category (Table 9). Accordingly, the total HEP value was obtained equal to 0.037 for this operator according to Eq. (5).

The CPCs analysis pertaining to the supervisor control shows that CPC_4_ and CPC_8_ have a negative effect on performance reliability. The results of negotiation with the operators showed that there were some problems in utilizing the procedures/plans at play. These problems can be referred to as (a) the lack of any procedures/plans for some special cases, and (b) difficulty in having access to some procedures/plans (CPC_4_) in some cases. It was also found that “adequacy of training and experience” (CPC_8_) has some shortcomings in practice. For example, the training programs do not suit the operator's working needs. In addition, some of the operators lack sufficient experience to perform their assigned job. In this light, the probability distribution of the control mode is varied for this operator so that scramble (0.01), opportunistic (0.38), and tactical variations (0.60) were placed in this category (Table 9). Accordingly, the total HEP value was calculated to be 0.03 for this operator based on Eq. (5).

The CPCs analysis relating to Boardman operator shows that CPC_5_, CPC_6_, and CPC_9_ have had a negative effect on performance reliability. Since CPC_2, 3, 4_ are dependent on CPC_6_, reduction of the available time (CPC_6_) can be affected by these CPCs. “The crew collaboration” (CPC_9_) is also inefficient here, which can be attributed to the competitiveness and job insecurity, the overlap between the official and unofficial structure, the level of distrust, etc. The poor collaboration among the operators, in turn, would put the working conditions in an incompatible mode, and might make MMI and operational support, and the availability of procedures inappropriate. Therefore, the probability distribution of the control mode in these conditions is varied for this operator so that scramble (0.04), opportunistic (0.59), and tactical variations (0.36) could be put in this category (Table 9). In view of that, the total HEP value was calculated to be 0.08 for this operator as per Eq. (5).

The CPCs analysis pertaining to the outside operator shows that CPC_4_, CPC_5_, CPC_8_, and CPC_9_ have a negative effect on performance reliability. The CPC_2, 3, 4,_ are dependent on CPC_5_, and CPCs_1, 8_ are dependent on CPC_9_. Similarly, in the analysis of the tasks relating to the outside operator, it was found that “the availability of the procedures/plans” (CPC_4_) was inappropriate, “the number of simultaneous goals” (CPC_5_) was beyond his actual capacity, “the adequacy of training and experience” (CPC_8_) was inadequate, and “the crew collaboration” (CPC_9_) was insufficient. As mentioned earlier, the requirement for the conduct of several simultaneous tasks would put “the conditions of working” (CPC_2_) in an incompatible fashion, and could bring “the adequacy of MMI and operational support” (CPC_3_) and "the availability of procedures" (CPC_4_) to an inappropriate state. Therefore, the probability distribution of the control mode in these conditions is varied for this operator so that scramble (0.11), opportunistic (0.76), and tactical variations (0.13) could be put in this category (Table 9). The total HEP value was obtained as equal to 0.09 for this operator based on Eq. (5).

The results of this study are, to a large degree, consistent with those of Yang et al.’s [1] study. They conducted a study on a motor tanker that was discharging crude oil at Karachi oil terminal. The identified CPCs that were involved in the accident consisted of the incompatible working conditions, inappropriate availability of procedures/plans, inappropriate adequacy of man–machine interface and operational support, the number of simultaneous goals beyond the actual capacity, continuously inadequate availability time, inadequate adequacy of training and experience, and deficient crew collaboration quality. Therefore, the results of this study are consistent with those reported by Yang et al. [1] while the current study's structure is easier than that of Yang's study. The reason for this is that collecting information about CPCs, converting the discrete data (Table 9) to the continuous date and analyzing them based on Bayesian approach using MSBNx software is easy in the current study.

Table 10 also compares the information associated with the basic and Bayesian CREAM methods together. As it can be observed, the CPCs level and control modes are discrete and deterministic (either 0% or 100%) in the basic CREAM. On the other hand, the number of the CPCs with positive and negative effects will be counted and used to determine the control modes. However, the data pertaining to the CPCs level and control modes are semi-deterministic and very diverse in Bayesian CREAM. The CPCs level is determined considering the dependency of the CPCs. In this context, COCOM is also calculated in a continuous nature. However, the probability of the human error calculated by Bayesian network is slightly higher than what was obtained for basic CREAM. This means that there is a consistency between basic and Bayesian CREAM and thus the validation of the current study is partially confirmed. Moreover, the reliability of the CREAM BN was previously tested by Kim et al. [9].

## Conclusion

Statistics show that human error is a key factor contributing to the occurrence of more than 80% of chemical and petrochemical accidents [18]. Therefore, human error analysis with the aim of the quantitative estimation of human error probability is a crucial undertaking in these industries. For this purpose, CREAM was utilized as one of the second-generation HRA techniques that is widely used in various industries, including the petrochemical industry. There are, however, significant shortcomings in traditional CREAM pertain to specifications of CPCs and the estimation of control modes, which are at play as two core concepts in HEP calculation. Therefore, it is recommended that other researchers also attempt to explore more detailed parameters associated with each CPC and identify the rate of dependency among them (especially, the CPCs with neutral effects) in various case studies in order to overcome the mentioned shortcomings. Then, the effect of the CPCs with new parameters should be evaluated on the HEP. In addition, it is suggested that managers improve the CPCs with the negative effects in order to reduce HEP and increase human performance reliability. Reduced HEP, in turn, can lead to a significant reduction in the occurrence of accidents. This motive can convince enough the managers to focus their attention on such recommendations.
